# The making of pastoralisms: An account of the Gaddis and Van Gujjars in the Indian Himalaya

**DOI:** 10.1186/s13570-022-00259-z

**Published:** 2022-10-24

**Authors:** Raghav Srivastava

**Affiliations:** Yale School of the Environment, 195 Prospect St., New Haven, CT 06511 USA

## Abstract

The pastoral communities of the western and central Himalayas have, for centuries, presented the modern Indian state with a problem of governance (as it has often been projected). Their existence, largely outside the domains of fixed property and capitalist production relations, has long since been problematized. Their seasonal migrations and vertical movements in space and time have enabled neither a smooth nor complete assimilation of these peoples into one of the state’s existential imperatives—the sedentarized market economy. The interventions imagined and imposed in response, have largely shaped these unbalanced relationships which, I assert, closely follow the features of James Scott’s high-modern state projects (Scott 1998, Seeing Like a State: How Certain Schemes to Improve the Human Condition Have Failed). Through this, an articulation of the tension between the Indian state and two transhumant pastoral communities—the Gaddis and the Van Gujjars—will be attempted to be juxtaposed and contrasted. I will attempt to show how the state in its various forms has used an array of legitimizing arguments and tools—morality, conservation, revenue, development and climate change—to ‘settle’ the Gaddis and Van Gujjars out of their traditional roles, into a lifestyle more conducive to measurement, surveillance and control: a ‘de-pastoralization’ of the pastoralists (Caravani, J Peasant Stud 46:1323-1346, 2019), towards the larger statal goals of assimilation, measurement and appropriation (Foucault 1995, Discipline & Punish: The Birth of the Prison). Through this, the changing and seemingly haphazard dynamics of legitimization will be attempted to be situated in their contexts and used to characterize the contrasting situations of both these communities—while highlighting the need to complicate the role of their social and religious identities in the making of their pastoralisms.

## Introduction

This paper will focus on the ‘pastoralisms’ of two transhumant pastoral communities—the Gaddis of Himachal Pradesh and the Van Gujjars of Uttarakhand. Their existence, on the periphery of fixed property and capitalist production relations, has long since been problematized by the high-modern capitalist states that have governed them—given their existential imperatives of measurement, surveillance and control (Foucault 1995; Scott 1998). The seasonal migrations and movements of these communities in space and time have consistently obstructed their assimilation into another of the state’s existential imperatives—the sedentarized market economy. The interventions imagined and imposed in response have largely shaped this unbalanced relationship—and contributed to the making of distinct patterns of pastoralism. I also use the term in plural, and I will assert that each of their pastoralisms is indeed unique in their making. To reduce them to a single term is, I believe, to eliminate much of their meaning.

In this paper, I will draw upon existing literature and expressions of state intent (policies, laws and court orders), to show the use of an array of legitimizing arguments—morality, revenue, conservation and (most recently) climate change—in an attempt to ‘settle’ the Gaddis and Van Gujjars out of their traditional roles, into a lifestyle more conducive to measurement, surveillance and control. The changing and seemingly haphazard dynamics of legitimization, and state attempts at sedentarization, will be attempted to be situated and distinguished in their contexts. I wish to neither describe nor study their pastoralisms dominantly in terms of culture and symbols (Wagner 2013) nor in terms of techno-quantitative resource use and biophysical adaptations (Bhattacharya and Sathyakumar 2011). I am also not qualified enough to do either.

I will attempt to bring out the localized contexts within which their ‘pastoralisms’ are enacted. I will also contrast the dynamics of the state’s relationships with each of these two communities and attempt to situate their differential ‘treatments’ in their political and socio-economic environments. I use ‘environment’ here in the sense that Wagner (2013) critically adapts for her ethnographic work with the Gaddis, as a ‘process’ which is conceptualized beyond the nature-culture dualism. I will draw upon Wagner’s narrative methodology in her ethnography of the Gaddis, in attempting to straddle the extremes of romanticizing (which is the net effect of according it a higher cultural and ecological value) and essentializing (which comes from viewing their transhumance in terms of resource use and adaptation) their lifestyles.

There is perhaps an over-assignation of individual and community agency in Wagner’s observations, at the cost of granularizing certain material/functional factors, like local market relationships and the construction of religious identities, that contribute to the highly differentiated situations of these two neighbouring pastoral communities—something Gooch (2004) does mention in her own work with the Van Gujjars. I will attempt to bring out some of these functional constraints and show how they have also been contributory to the coming about of the distinct ‘pastoralisms’ of the Gaddis and the Van Gujjars.

The contexts of the Gaddis and Van Gujjars (who I will interchangeably refer to as ‘Gujjars’ throughout this paper—not to be confused with the larger pastoral community of the northern and western Indian subcontinent), as per documented histories and linguistic studies (Das 2000; Rahi 2020; Dhiman et al. 2022)—have been highly distinct in their making and unfolding. Instead of discussing them separately, I would rather present them as part of a singular narrative to better bring out the distinctions. Although this method may suffer from the vice of appearing contrived at times (in an attempt to contrast), I will only do as much as to state what has already been perceived and seen. I do so in the hope that this method will be useful in creating a dialogue between two worlds (of the Gaddi and the Van Gujjar), which in some senses exist in a high degree of intimacy (in terms both of geography and resource utilization patterns), but which rarely occur together discursively. I hope also to bring about this integration in the image of the ‘ecosystem approach’ Tarnowski (1997) alludes to in his analysis of the evolution of perspectives on Himalayan pastoralism, which—he argues—have suffered from a studious disability of seeing elements of change as discrete and somewhat disconnected from their total systems. This is analogous to, but distinct from ecological science’s ‘ecosystem approach’ (Rowe 1992), and refers to a broader ecosystem which encompasses all of the elements—natural and unnatural, material and affective, given and changing, and assumed and imposed—that are involved in the making of their environments. This includes their historical contexts, their community identities and their socio-political situations.

## Methodology

This article is primarily a critical discourse analysis based on a review of existing literature. For setting theoretical bases, upon which I make these assertions, I will rely on secondary sources—ethnographic and sociological studies done within these communities, as well as other anthropological literature on Himalayan ecology, on other transhumant peoples (like the Bakerwals of Jammu and Kashmir), and political-philosophical theoretical discussions. I will also rely on my own experiences as a lawyer and a practitioner in the conservation ‘industry’ in India and my work with the United Nations Development Programme’s SECURE-Himalaya Project^1^ in the states of Himachal Pradesh and Uttarakhand. To make the arguments themselves, I will rely primarily on (i) conversations and interactions with Praveen Kaushal of the NGO ‘SOPHIA’ (Society for the Promotion of Himalayan Indigenous Activities), who has been working with the Van Gujjars to help secure their livelihood and belonging for over thirty years; (ii) data from the existing aforementioned ethnographic studies; (iii) laws and policies which interact with these communities; and (iv) judgments and pronouncements of higher (Constitutional) courts of India.

I will punctuate this section with an acknowledgement of my own situation in this work. I approach this paper out of academic interest and a sense of geographical and social fraternity with the Gaddis and Van Gujjars. I have been exploring the Western Himalayas on foot, sporadically, since 2015. It was only until much later that I realized most of the trails I had trodden had been made by these two communities in particular. The irony, of their own trails having contributed to the epidemic of aesthetic, upper- and middle-class ‘environmentalism’ in India (Mawdsley et al. 2009), which has become another source of disruption to their environment-making, was not lost on me. I have chosen to compare their situations owing to (i) the ubiquity of use of their historical trails for recreation and tourism across the hill states of Himachal Pradesh and Uttarakhand and (ii) the evident contrast between their pastoral situations, despite their geographical-cultural proximities. I acknowledge that I approach this study from a certain cognitive and epistemic distance, which must be a caveat to the conclusions and observations I make.

Having worked as a practitioner in biodiversity conservation (also since 2015), I am acutely aware of the structural and discursive contradictions which are characteristic of that sector and write out of a sense of allegiance to that cause as well. Lastly, I write this paper as an Indian, upper-middle-class, *savarna* Hindu male enrolled at Yale University, with all of the attendant privileges that come from this amalgam of identities. I have been able to gather and access information and data which would have been difficult for persons who may not have enjoyed my privileges of access and make this a potentially worthwhile (if I may say so myself) output of desk research. Like all others, I have not been immune to the more general, as well as more context-specific, affective residues of the COVID-19 pandemic: having had one foot each in two continents, in navigating the requirements of my master’s program, whilst attending social engagements and duties which were required of me as a person, a lawyer, a citizen of my country and as a son. In spite of my best efforts, these have had a non-negligible impact on my work.

## The contexts of Gaddis and Van Gujjars

The historical contexts of the Van Gujjars are less documented than that of the Gaddis and have been largely sourced through informal and ancillary sources. They are thought to have come originally from Kashmir, via Sirmaur, to the *Bhabar* regions of the Shiwaliks, about 1500 years ago (Benanav 2015). Gaddis, in contrast, have better-documented histories going as far back as 700 CE (Chakravarty-Kaul 1998; Saberwal 1996a). Both, however, also have well-recognized and documented mythologies which situate them as belonging in their geographic contexts—the Gaddis as shepherds of *Shiva*^2^ (Wagner 2013) and the Gujjars as either descendants of guests of a local *raja* (who developed a taste for their milk and invited them into the plains from Kashmir) or the progeny of one (Benanav 2015). This is especially typical of Hindu communities in India, in that their mythologies are imbued with a legitimizing concern—through a claimed intimacy with either deity or royalty.

The other records which give some insight into their historical situations are official correspondences from the late nineteenth century—following the British appropriation of forests (Gooch 2009), and the extension of British law and bureaucracy over them.^3^ In this way, what had earlier been marginal to the government in the subcontinent, was appropriated by the modern British state to contribute to its production of surplus value. Not only was the economic value of the forests’ timber of interest, but the extension of the administration itself created value for the state as well. The British state assumed, in a sense, the part of middlemen between the pastoralists and their use of the forests, through the use of theatrical tools: individual permits, official letters and laws (Gooch 2004; Saberwal 1996b). These created their authority as much as they represented it.

The case of the Gujjars was distinguished even then—being as they were ‘full pastoralists’ who possessed neither a ‘permanent house nor cultivated land’ (Gooch 2009). Creating intruders out of them was the path of least resistance given the colonial state’s goals. Constructing the Gujjars as outsiders was politically simplified by their total lack of adherence to the sedentarized, appropriation-appropriate life of the ‘normal’ subjects of the state. An extract of a government order from the North-Western Province, issued in January 1885, provides evidence of these relational dynamics between the Gujjars and the colonial state (Gooch 2009: 242):

1. *All Gujars and other wandering herdsmen are warned that for the ensuing year, only 150 heads of buffaloes will be allowed to graze in the Jaunsari, Tehri Garhwal, Raimgarh and Dadi partitions of the Jaunsar Division.*
2. *As regards the Basbarh partition of the Jaunsar division, 500 heads of buffaloes will be permitted to graze there.*
3. *The owners of cattle who wish to graze in the Jaunsar and Bashabar forests during the current year should apply for grazing passes at the office of the Dy. Conservator of Forest Jaunsar Division, Dehra Dun, between 15 February and 18 March.*
4. *The 150 heads of cattle in the first para will proceed to their grazing grounds via Chakrata road and the Landsroos, and those going to Bashabar will proceed up the Giri River.*
5. *No cattle will be permitted to proceed up the Tons or Jamuna rivers and police guards will be stationed at Sangola Bridge to prevent Gujars from doing sv* [sic]*.*
6. *Any Gujar attempting to traverse Jaunsar without a pass or proceeding by a route not laid down in the passes issued at Dehra Dun will be liable to severe punishment and will be compelled to return to the plains.*
7. *The usual grazing fees will be paid by the Gujars 1 month after reaching the grazing grounds.*
8. *The Gujars will return to the plains by the same routes they followed when going up*.

In his seminal work *Seeing Like a State*, James Scott deconstructs the methodologies of ‘high-modern’ state projects, positing certain common characteristics that are found across a wide spectrum of statal processes since the late nineteenth to twentieth centuries (Scott 1998). The restrictions contained in the order reproduced above are in the image of Scott’s conceptualization of the abstracting, utilitarian logic of the rationalizing state (Scott 1998), whereby the exclusion of competing users of ‘timber’ and ‘forest-produce’—the objects of state protection, in the language of the state—became rationally necessitated. But a method of exclusion cannot be operative without a method of sanction.

Discipline and punishment, as Foucault (1995) so aptly titled his work, go hand in hand. Writing about England and other European penal systems of the time, he described the eighteenth and nineteenth centuries as a period where *From being an art of unbearable sensations punishment* [had] *become an economy of suspended rights* (Foucault 1995). The theatre of ‘retributive justice’ was in the process of giving way to the use of punishment as both a tool and arena of the extension of state control. Foucault’s analysis of the guillotine as symbolic of the trend towards homogenization and abstraction of punishment is particularly significant, and in conjunction with Scott’s theorization of how high-modernist ideologies are enacted by states (Scott 1998), provides a good framework for understanding how the British and Indian states addressed (and continue to address, as I argue subsequently) their ‘pastoralist problems’. While the colonial state transferred its authority and power eventually to Indian peoples, its objectives did not change significantly. A generalized and brief description of this change would be from expropriation and surplus creation of the British colonial state to appropriation and surplus creation within Indian political boundaries. A more detailed discussion of ways in which laws and policies are employed as instruments in legitimizing and effecting this ordering of multiplicities will follow in the subsequent section.

Their enactments of ways of self-identification also exist as an area of a distinct variety. The Gaddis self-identify as such themselves. The community identities are based in part on their historical and mythologized contexts, which is reflected in their narrative situation and integration into the general caste structure of the dominant agricultural communities. The Gaddis have aligned their own castes/*varnas* with those of the settled Hindu *Brahmans* and *Rajputs* of the regions of Bharmaur and Kangra in Himachal Pradesh (Wagner 2013), where the Gaddis are chiefly settled (Wagner 2013). Their dialect is also similar to those of Bharmaur and Chamba, and Gaddis are generally conversant in *Pahari* as well which is spoken chiefly in Kangra (Wagner 2013). This higher degree of linguistic integration has also played its part in shaping not only their community identities *vis-à-vis* the other residents of these regions, but also their political situations—which are, as I argue throughout, intimately involved in the production and reproduction of their pastoralisms.

The dialect of the Gujjars recalls the story of their long historical journey from Jammu and is a blend of *Dogri* and *Punjabi* (Benanav 2015)—which sits incongruously in the Hindi-speaking and highly sanskritized regions of Uttarkashi, Dehradun and Haridwar. Moreover, the Van Gujjars only became reified in their own referencing, as such, quite recently. They were known variously as *Jammuwallahs*, *Dudh* Gujjars (Gooch 1992), and among themselves identified as just Gujjars/Gujjaris. The *van* prefix (meaning ‘of the forest’) was only added in the 1980s, when their representative *lambardars* and *samitis*, assisted by an NGO called the Rural Litigation and Entitlement Kendra (RLEK), were involved in a movement agitating against the proposed eviction of Gujjars from their traditional winter *khols* in the Shiwaliks (Benanav 2015; Gooch 2009). The state-legitimized reason behind this dispossession was the proposed notification of the Rajaji National Park (now Rajaji Tiger Reserve) in the area, under the recently enacted Wild Life (Protection) Act of 1972. This situation, discussed in greater detail in the next section, marked another point of inflection in the negotiation of the Gujjar identity *vis-à-vis* a new institutional constraint which had been added to their environment-making. In the face of new legislation to back the century-old claim of the state, with no real evidentiary basis, that the Gujjars’ pastoralism was detrimental to the ecology and health of the forest, the Gujjars had to emphasize their identity as ‘friends of the forest’ (Gooch 2009): the pastoralists who had always lived in harmony with their forests and who had the ecological knowledge to ensure its sustenance. They had to precipitate this aspect of their identity: an ‘articulation’ in the sense that Li (2000) described in her study of tribal identities in Indonesia, as contingent and borne of the dialectics of relating to power structures against which they inevitably collide. It is significant to note that the entire discourse around the overgrazing and destruction of forests by Gujjar herds existed since the nineteenth century and acquired its legitimacy by virtue of that alone. The discursive knowledge produced therein and thereby has been in response to a claim of *power*, not fact.

Van Gujjars have a more homogenous community and are less stratified socio-economically (Gooch 1992) as compared to Gaddis (Wagner 2013), in part perhaps, owing to the historical absence of a surplus appropriation, where even depositing money in banks for interest is traditionally looked down upon in their Islamic tradition (Gooch 2009). From a more phenomenological perspective, their community and religious structures also merit mention as participating in the making of this distinction. Gujjar ties of kinship remain strong and internally enmeshed, in contrast with the Gaddis who have settled in Kangra and Dharamshala in Himachal Pradesh, where their intermarriages with *Paharis* and other folk are common (Wagner 2013). The Van Gujjars’ status as religious minorities (Muslim), ethnic minorities (tribespeople) and politically marginalized has also likely been contributory to the making of a greater need for reliance on their own people—although there exist internal structures and traditions within them that more than account for this, such as endogamy and other kinship ties (Gooch 2009). I would argue, however, that Gooch has accounted for these to explain *why there exists a strong internal solidarity* within the Van Gujjars and not *why there does not exist a more diffused fraternity or kinships, with the other communities* with which they interact.

In terms of their political situations as well, there is something to be said of the differentiated processes of Gaddis and Gujjars. This again is a function of the interplay of variously articulated identities. The first which has frequently found passing mention in socio-anthropological studies (Gooch 1994; Gooch 2009; Nusrat 2011) is that the Van Gujjars are Muslims. In this sense, they are not only ‘othered’ from the majority of the Hindu-dominated states between which they move (Uttar Pradesh, Uttarakhand and Himachal Pradesh), but also from the other Gujjars^4^ of north-western India, who are predominantly Hindu. Axelby (2016) has written about the Gujjars and Gaddis in terms of their rights and access to the property in Himachal Pradesh. There are, however, certain distinctions to be drawn—Axelby’s observation, for example, that the Gujjars are classified as a Scheduled Tribe does not hold in their ‘home’ states of Uttar Pradesh and Uttarakhand (Axelby 2016). In this and other works on the Gujjars, moreover, the role of the religion has not been analysed more deeply in the making of their pastoralism.

Singh, writing about the homogenization of Islamic identities in South Asia, criticizes this oversight (2012). He noted that in the few works where Gujjar religion has been recognized by its authors as relevant and revelatory, it is often conflated with their ethnicity and not looked upon as distinct from it (Singh 2012). Singh draws upon prior ethnographic and religious studies of the Gujjars with a view to better parse their relationship with the dominant Islamic discourses of their time and space. He addresses the under-complication of the Gujjar’s Islamic identity in academic discourse and offers a critique of more secular discursive methodologies which have been preferentially used in the study of marginalized communities in India. He points to the fact that the adjacent districts of Saharanpur and Muzaffarnagar are the ‘heartland’ of the Islamic Deoband School,^5^ which overlaps with where the Van Gujjars have traditionally set up their *deras* for the winters (Saharanpur and Dehradun districts, chiefly). Singh notes that the Deoband school, to which most *madrasas* in north India are affiliated, did not display much interest in spreading its influence to the Van Gujjars (Singh 2012). He theorizes that they converted to Sufi Islam at some point in the thirteenth to fourteenth centuries CE but also notes that they have not really attracted the attention of Islamizing movements, in the universalized nature of proselytizing attempts to assimilate fringe groups into the dominant version of the religious practice (Singh 2012).

Highlighting the political marginalization of the Van Gujjars is not to say that the Gaddis have not been marginalized. As pastoralists, they have also negotiated and re-negotiated pressures from the state and its attempts to restrict and control their mobility (Chakravarty-Kaul 1998; Saberwal 1996a; Wagner 2013). This marginalization has been limited, however, by some sharp differences (vis-à-vis the Gujjars) in their social and material relationships with their political geographies. Up till the eighteenth to nineteenth century CE, there seems to have been an encouraging and inclusive relationship of the kingdoms of Chamba and Kangra with the Gaddis (Chakravarty-Kaul 1996; Saberwal 1996a; Saberwal 1996b). Chakravarty-Kaul (1998) and Saberwal (1996a) both note that their customary usages/allowances^6^ prior to the imposition of colonial strategies of exclusion and control were founded on the recognition of reciprocities. The *rajas* recognized the value that the Gaddis’ pastoralism added, in converting the remote and inaccessible grasses of the *dhars*, to wool, milk and meat for their societies (Saberwal 1996a).

With the consolidation of British colonial power in north India in the nineteenth century (following the annexation of Punjab in 1849), the objectives of state policy changed, which prompted a change in administrative strategies and discursive methods of disciplining. There were overlaps with the Gujjars’ situation described earlier (the Indian Forest Act was central law, applicable to all British-controlled provinces)—they were assigned fixed routes of migration from which they could not diverge (Saberwal 1996b). In addition, the discourse of pastoralists as destroyers of forests was pushed in the case of Gaddis as well, articulated through policies such as dual taxation (on both usage of pastures, as well as on the migration itself—ostensibly to discourage goat grazing), and a requirement to move at least 5 km every day (Saberwal 1996b). Gradually, Saberwal (1996b) and Chakravarty-Kaul (1998) both note that their customary usages were eroded under the shearing force of the exclusionary colonial institutions and discourse.

With respect to the Gaddis, Saberwal (1996a) has noted a degree of political influence which they wield in Himachal Pradesh post-independence, which he attributes to their statistical significance as a vote-base. What he does not allude to is the causative value of their status as a Hindu (i.e. majority) community and assimilation into the case structures of their settled neighbours (Wagner 2013), which stands out in distinction to the state responses to the Gujjar pastoral situation. Their political influence was visible in the shifting policies of the Forest Department consequent of interventions by elected representatives of Kangra, Kinnaur and other areas where Gaddis were settled (Saberwal 1996b). The report of a Grazing Advisory Committee constituted in 1972 provides evidence of this—which recommended a moratorium on the implementation of grazing restrictions for a 5-year period, as well as the opening of reserved forests for Gaddi grazing (Saberwal 1996b). Their political de-marginalization post-independence was also visible in their building and use of social networks to obtain surplus forage in winters from the surrounding farmlands of Punjab, Uttar Pradesh and Haryana (Saberwal 1996b).

An embodied sense of belonging, which is tied closely to their socio-political identity as Indian citizens/Scheduled Tribes^7^ of their region, is differentially held—in the Van Gujjars, it is more discernible as tied to the forests. Although the *van* in their name is relatively recent, their sense of belonging to the forest is not (Gooch 1992). But although Muslim Gujjars have been recognized and notified as Scheduled Tribes by the erstwhile Indian state of Jammu and Kashmir, and ‘Gujjars’ have also been notified as such in Himachal Pradesh, it is telling on the part of the states of Uttarakhand and Uttar Pradesh that they have not imparted this recognition to the Van Gujjars. I assert in the next section that this has been strategic in the theatre of delegitimizing their claims to rights as forest-dwellers.

### The role of post-colonial state policy and environmental discourse

In both Gujjar and Gaddi cases, top-down restrictions which were imposed by the colonial state as part of its strategies to appropriate exclusive use over forests have followed Scott’s theorization of the practical working of state efforts towards homogenization and legibility (1998): they have been pragmatized over time with the *metis* of locally negotiated informal concessions for loping, grazing, etc. (Saberwal 1996a; Chakravarty-Kaul 1998; Gooch 2009) These negotiations have in a large way influenced the temporalities and routes of their migrations since the late 1960s. Gooch (2009) has described these relationships in the case of the Gujjars as transactional and contingent and subject to what has become a systematic payment of cattle heads or bribes, which was evidently founded less on the magnanimity of the state forest bureaucracy. In the case of Gaddis, these relationships have been mediated largely through political representatives intervening on their behalf to create exceptions to existing exclusionary policies and laws (Saberwal 1996b)—which are not effected as amendments to laws and do have some of the features of local informality, informed by *metis*.

Following from this, it is not difficult to imagine that Gaddi and Gujjar pastoralisms are a function of their legal identities as well—which differ on paper in terms of the letter of law and policy, but even more so in the way that the temporalities of political and legal discourse constantly interact with these (laws and policies). Their legal identity is in that sense, also, like their larger environment, a verb rather than a noun (Wagner 2013). They are in a state of flux, being negotiated and re-negotiated with the state, which is now firmly entrenched as a mediator between them and their biophysical environments. The story of their pastoralisms is, in this sense, circumscribed at each point of inflection, by their respective politico-legal narratives—that have been as constraining as the anoxic air and bitter cold of the high mountains, as the heat and density of the foothills, and have perhaps not been situated as importantly as they should have, in the making of Gaddi and Van Gujjar pastoralisms.

The dominant discourse of forest management has changed the world over, and it is no longer the fashion to claim economic logic alone for preserving and conserving forests (even if that is still, as in the present Indian political situation, the effective net consideration). In India, even as early as the National Forest Policy of 1952 (the first of independent India), a recognition of the ecological and social value of forests was present—albeit to the extent that it did not interfere with the use of forests in the ‘national interest’, which is used euphemistically in Indian law for the exercise of state discretion (although both produce very different meanings). The National Forest Policy of 1988, which is the most recent version, embodies the contemporary discursive dominance of conservation in forest management and states at the outset as the basic objectives which *should* govern forest policy—restoration of ecological balance, conservation of natural heritage and meeting the fuel, fodder and other needs of rural and tribal populations. The *should* is revelatory—it presents all of these objectives as avowed and idealizes them to an extent—placing them beyond the practical limits of political reality. It is also relevant to note that policies in Indian law are not justiciable.^8^ True to character, in its schizophrenic pursuit of ends within a high-modernist ideological framework (Scott 1998), the Indian state still retains the colonial-era Indian Forest Act of 1927 without any substantive changes, as the operational logic of its forest management.

Although India ratified the Convention on International Trades in Endangered Species (CITES) in 1976, its Parliament had already signalled its uncritical subscription to the exclusionary model of ‘fortress’ conservation a few years prior, in enacting the Wild Life (Protection) Act of 1972. In a country where a large part of the marginalized population (largely tribal communities) was still socio-economically dependent on forests (Gadgil and Guha 1994), this law and the exclusionary forest categories which were created under the Indian Forest Act seem quite incongruous and contradictory to their avowed purposes. When understood for their real actual purpose, however, which was to extend state control and legibility in ways which were up-to-speed with the linguistic proprieties of international environmental discourse, they make more sense. Under the Wild Life (Protection) Act, for example, the creation of protected areas offers a one-time ‘settlement’ of the rights of existing forest users (decided unilaterally by the bureaucracy) and does not envisage them even entering the boundaries of the area thereafter, without the permission of its management.^9^

With regard to the Gujjars, their tensions with this modern avatar of conservation began in 1983—with the Uttar Pradesh Government notifying its intent to create Rajaji National Park, for which purpose the Van Gujjars would be evicted from their winter camps (Gooch 2009). The official discourse since colonial times—of branding them variously as nuisances and encroachers and creating a zero-sum narrative where recognizing customary rights for the Gujjars meant less rights for the villagers (Gooch 1992)—required no further substantiation for their exclusion from the proposed park to be accept as a pro-conservation strategy in the language of the state. No evidence or study was required to determine that their presence in the park would be detrimental to its ecology. On the other hand, according to Praveen Kaushal of SOPHIA (‘Praveenji’), the resettlement policies of the state are far more detrimental to the survival of the forests. The Van Gujjars have been allotted lands in the settlements of Pathri and Gaindikatta, near Haridwar. They have been allotted small plots and standardized two-room tenements per family—without any regard for their family sizes and how they make their living spaces (P. Kaushal, personal communication, November 25, 2020). Furthermore, only those families who shifted prior to 1994 have been given agricultural land for cultivation, which is largely infertile (Nusrat 2011). They are compelled to still maintain their herds for subsistence, and the enforced lack of mobility means that the buffaloes must be fed from the same area, year-round—increasing the pressure on the adjacent forest. In Praveenji’s view, it is not the Gujjars who are intrinsically nomadic, as much as the buffaloes. A policy which did not recognize and address the unique needs of the buffaloes was apt to result in disaster, as it has.

The Gaddis fared better in terms of their qualified, but continued, access to the forests of Himachal Pradesh post-independence. However, for them too, all is far from straightforward. The Scheduled Tribes and Other Traditional Forest Dwellers (Recognition of Forest Rights) Act was enacted in 2006 (‘Forest Rights Act’) by a Parliament which purported to correct the ‘historical injustices’ which forest-dwelling Scheduled Tribes, and other forest dwellers, had faced at the hands of the state. Lofty objectives, but the schizophrenia of the high-modernist state was not to be lost here—the Ministry of Tribal Affairs has, uniquely, been *explicitly* assigned a nodal role in the implementation of the Forest Rights Act. The Act, by virtue of the people it seeks to benefit, would require a government agency to have a presence in the hinterlands and forests, where the forest villages and tribes are. The state tribal departments did not have this, but the forest departments did. The forest departments have thus become the *de facto* authorities under this law and are charged with assisting in the making of claims to community forest management and tenurial security to the use of forests for subsistence—while also charged with managing the same forests and protected areas, to the exclusion of these same people and uses.

Uttarakhand and Himachal Pradesh are among the worst performing states in India, in the recognition and settlement of forest rights (Ministry of Tribal Affairs, Government of India 2019). On an official visit to Uttarkashi^10^ in 2019, as a legal consultant working for UNDP on the SECURE-Himalaya Project, I asked the District Collector during a meeting about the status of forest rights in the district. In reply, I was told that the people were ‘not interested in filing claims’ and that only a few spiritual gurus had been granted individual titles for their *ashrams*. The Gaddis are slightly better off than the Gujjars, in that many of them do have privileges and concessions recognized under the Indian Forest Act, 1927 (Proceedings of the Himachal Pradesh State Level Monitoring Committee, June 2019). These are, however, in the nature of allowances/privileges, and lack the security of tenurial rights.

In Himachal Pradesh, Himdhara (a rights-based environmental action collective) has been actively involved in the struggle for better implementation of the law. As per their experience, a lack of political will and the insecurity of influential persons who have grabbed common lands in or near forests are chief among the reasons for its non-implementation in Himachal Pradesh (Himdhara 2018). Forest dwellers who are not Scheduled Tribes (like the Gujjars in UP and Uttarakhand) require to prove their dependence on the forests for three generations or 75 years. The discretion of what would count as valid evidence rests with the de facto authority which is the forest department. Although there is ample guidance and precedent to guide the forest department to be contextually sympathetic in interpreting what is acceptable as evidence (Srivastava 2019), they often choose to disregard claims on the basis of the lack of documentary evidence (Gooch 2009). Their only documents are grazing permits, which were issued to a few families, decades ago, whose names no longer match those of their holders. These do not meet the department’s unwritten standards, as Praveenji confirms. Actions and omissions like these, which appear systemic, unintentional and normalized, are commonly employed to further the high-modernist state’s goals of maintaining control over its forests, while also preventing the efflux of power and resources away from those to whom it is invariably beholden.

The Himalaya has in recent times become a focal point of concerted efforts and funding, to mitigate and adapt to climate change. It represents a unique concentration of biomes, biodiversity and cultures and is known (along with the Tibetan plateau) as the ‘third pole’ for the many glaciers within it, whose retreat has become powerful affective imagery of the evidence of the global warming that is already catastrophic, but only sporadically spectacular (Morton 2011; Nightingale and Rankin 2015; O’Neill and Smith 2014). The structure of policy formulation and decision-making in the donor and implementing development/conservation organizations continues to follow the same top-down approach from a studious distance, like that of the state (Blaikie and Muldavin 2004).

The SECURE-Himalaya Project in India, a joint venture of the UNDP and the GEF, is emblematic of this approach. The project document stands in wry contradiction to Blaikie and Muldavin’s optimistic view (2014) that the Theory of Himalayan Degradation has achieved obsolescence in environmental discourse. To quote from its description of the threats to snow leopard populations in India, “ … The increasing snow leopard-human conflicts is likely a manifestation of habitat degradation due to over-grazing and over-harvesting of natural resources by humans and their livestock.” (UNDP 2017: 10). Hussain’s excellent and incisive anthropological study of snow leopard conservation provides a useful critique of this broad approach to conservation in the Himalayas (2019). He problematizes the assumption that snow leopards preying on livestock is ‘unnatural’, which, like much of environmental discourse, relies on its historicity for legitimacy, rather than actual data. He points to studies, including his own research, which indicate that livestock predation is common even in areas of high ‘natural’ prey base densities (Hussain 2019).

Facts are tailored to arguments, rather than the other way round, which are political and value-laden: such as the assertion that the snow leopard is a keystone species (which is taken as a premise of most conservation research, but rarely if ever as a question) (Hussain 2019). This kind of discourse represents the privilege of western aesthetics of nature and its preservation, in a framework of transcendentalist values and capitalist alienation, at the cost of essentializing local livelihoods and identities. Hussain asserts that the livestock which form a substantial part of the prey-base of snow leopards (Hussain 2019; UNDP 2017) represent a subsidy to its existence by local pastoral populations. The narrative of a historical snow leopard population decline is also far more contested in terms of data than is presented in this narrative (Hussain 2019). The schizophrenia of high modernism is incarnate here as well—where the practice of pastoralism is accepted as having declined across the Himalayas, but is also claimed to be the single largest threat to the snow leopards (UNDP 2017). Among others, imagery of the *bugyals* (alpine meadows of Uttarakhand) has been employed in the Indian context (UNDP 2017), to create an affective connection with the ‘spectacle of (pristine) nature’ (Hussain 2019: 10), in this new discourse of climate change in the Himalayas.

There is a judgment of the Uttarakhand High Court rendered in August 2018 in the matter of *Aali Bedni Bagzi Bugyal Sanrakshan Samiti* v. *State of Uttarakhand* (Writ Petition No. 123 of 2014), based on a petition filed by a local conservation body, which sought orders for the protection of Aali, Bedni and Bagzi *bugyals*. Throughout the 60-page judgment, hyperbolic references are made to the *bugyals* in an almost obsequious fashion, with no basis in fact—*Bugyals are maintaining the ecosystem*. *The damage caused to the eco-system i.e. Bugyals is considered to be the key to global warming and the melting of glaciers* [sic!] (page 2). The hegemonic narrative of climate change appears to have been wholly internalized by this particular bench of the Uttarakhand High Court. It ultimately directed, among other things, the removal of semi-permanent huts which had been erected by the forest department, as well as a blanket ban on ‘commercial grazing’ activities, stating that *the local shepherds alone will be permitted to graze their cattle on the alpine meadows/sub-alpine meadows …* (page 60).

Another interim order of the same court (in a different case) in June 2018, castigated the state government for attempting to give the Van Gujjars ‘undue benefit from the state exchequer’ in the guise of rehabilitating a few families from Corbett Tiger Reserve (Upadhyay 2018). In June of 2020, when the matter of their rehabilitation from Rajaji Tiger Reserve continues to be *sub judice*, forest officials assaulted Van Gujjars following an interrogation and burned their settlements in the Asharodi forest range of the Tiger Reserve (Fanari 2020).

## Conclusions

In this paper, I have attempted to describe the differentiated pastoralisms of the Gaddis and the Van Gujjars, which have been made in their respective environments of discursive, socio-economic, political and biophysical constraints. I have attempted to describe these using existing theoretical frameworks, while not wholly subscribing to any one of them. I have attempted to fill what I saw as a gap in the study of these communities and tried to be sincere in my pursuit of a work which was more phenomenological, in the sense that it does not assign subjective values to any ideology, culture and biophysical relations in the making of their situations. I attempted to address myself to the various constraining aspects of their material contexts (at the expense, perhaps, of creating erudite abstractions which undoubtedly would have made for a better read). I have fallen short of this objective, though—there are issues which I have touched upon here, which merit a deeper and more dedicated investigation than I have given them. Through the paper, I have attempted to bring out how the various iterations of the state in India have used morality (in terms of resource sharing), revenue (taxes as a state prerogative), conservation (of high-altitude ecosystems, in the exclusionary model) and climate change—to differentially affect the pastoralisms of the more nomadic Van Gujjars, and the more agri-pastoral Gaddis.

The pastoralism of the Van Gujjars is liable to be romanticized as extra-market, or pre-market (Gooch 2009). While it was outside the confines of the sedentarized economy, they always occupied a specific niche in their regional economy and had a simplified catalogue of exchange—sell milk (in the lower altitudes) and milk products in the higher hills, and use the cash for the sustenance of them and their herds (Gooch 2009). Today, their integration into the sedentarized cash economy has become more complicated—and largely owing to a rise in their dependencies on it. Nusrat (2011) has studied and documented their barter transactions and found that they are inexplicable in terms of neoliberal economic logic i.e. the value of what they sell regularly exceeds what they receive. Do they make these exchanges out of desperation alone? Is a cash-based logic sufficient to describe their economic situation? Axelby (2016) avers to the economic viability of mountain pastoralism in the Western Himalayas, in spite of the many constraints and challenges to its pursuit, and characterizes it through the politics of ‘rights’ and ‘access’. Nusrat (2011) observes that the inaccessibility of their living spaces creates a greater dependence on barter for the availability of daily needs. Still others have studied sedenterization processes (in Uganda) as borne of larger contextual drivers, changing relations of production and gender (Caravani 2019). These are complex questions that, I would humbly suggest, may benefit from further research in the Van Gujjar and Gaddi contexts.

The Muslim Van Gujjars have not been the subject of a census so far (Singh 2012). The higher judiciary seems to be less sympathetic to their historical situation as well, as I have argued in the previous section. Crucially, in 2017, both Uttar Pradesh and Uttarakhand voted in Bharatiya Janata Party (BJP) governments^11^ which continue to govern these states. Through a combination of law, selective retributive penal actions, and communal electoral rhetoric, the villainization and othering of anyone with a Muslim identity are becoming increasingly normalized, particularly in the state of Uttar Pradesh (Singh 2020). Concurrently, the symbolic values and social meanings of Hinduism are also changing. In this context, it becomes imperative to investigate the religious articulations of both Gaddi and Van Gujjar identities and their role in determining their political and legal identities.

This paper was an attempt to describe the making of pastoralisms, but I realize that the eventual narrative presents a story of their un-making. The relentless pursuit of capitalist growth in high-modernist ideological frameworks, as I have attempted to show, seems to be deeply antithetical to the pursuit of justice, equity and welfare. I hope that the academic abstraction and inaccessible language which often conceals a broader understanding of these complex connections find less-obstructed expression. I take solace in the many movements that arise in response to these injustices, which represent to me a higher form of articulation than the written word.

## Footnotes

1 A joint conservation and development project of the United Nations Development Programme (UNDP) and the Global Environment Facility (GEF), to *ensure conservation of locally and globally significant biodiversity, land and forest resources in the high Himalayan ecosystem, while enhancing the lives and livelihoods of local communities* (UNDP 2017).

2 A Hindu deity, with roots in animistic traditions pre-dating Vedic Hindu religion in India.

3 The first (of the many) Indian Forest Act was enacted in 1878, shortly following the constitution of the Indian Forest Service.

4 So-called. The Bakerwals of Jammu and India-governed Kashmir are also believed to be a sub-community of Gujjars and, together with the *Banihara* Gujjars of the same region, are the other two distinctly Muslim Gujjar communities of northern India (Warikoo 2000).

5 One of the four major schools of Indian Islam, the Deoband school had its roots in the failed first war of Indian independence of 1857, following which the Dar-ul-ulum (the principal madrasa of this school) was established, its efforts aimed towards balancing a complex vision of a communally united anti-colonial front, while countering the Hindu right’s own cultural nationalist projects of the time (Singh 2012).

6 Chakravarty-Kaul (1998) has conceptualized these as customary usages per se, but they were also likely a product of sovereign allowances or permissions, sometimes also inclusive of *pattas* or titles to lands (Saberwal 1996a).

7 A legal designation created under a constitutional provision (Article 342), which was enacted as a form of ‘positive discrimination’, to bring into the political and social mainstream those tribes which had been marginalized or criminalized in pre-independent India. The Indian Constitution delegates the responsibility of identification and designation to the President, in consultation with the Governor (who acts on the aid and advice of the state cabinet of ministers) of the state in respect of which the tribe(s) is notified.

8 The Supreme Court of India has extended its interpretation of separation of powers in the Indian context, to preclude any kind of pronouncement on policies issued by the executive, unless they are wholly arbitrary or patently antagonistic to the Constitution. (See *Kalpana Mehta* v. *Union of India & Ors.* (2017) 7 SCC 295). In the current political milieu, the Apex Court has become even more conservative and even worryingly selective, in its willingness to interfere with arbitrary executive action (Trivedi 2020).

9 See sections 19, 27 and 35 of the Wild Life (Protection) Act, 1972.

10 The Gangotri and Govind Pashu Vihar National Parks, as well as many of the Van Gujjars’ traditional summer pastures, lie in this district. Much of their sales of milk and milk products are also made here.

11 The BJP has been the principal center-right party in national Indian politics since the late 1970s. Since 2014, it is arguably more right-wing and in line with the rhetoric of its ideological branch, the Rashtriya Swayamsewak Sangh (RSS). The RSS propagates a vision of cultural nationalism which identifies all those who do not follow Vedic Hindu religion, as outsiders and anti-nationals. The rhetoric is particularly antagonistic towards Muslims in post-colonial India. (Andersen and Damle 1987)
